# Aorto‐pleural fistula successfully treated by one‐lung ventilation and Endobronchial Watanabe Spigots

**DOI:** 10.1002/rcr2.382

**Published:** 2018-11-01

**Authors:** Takunori Hozumi, Koichiro Kajiura, Kei Nakamura, Haruki Taniguchi, Takao Goto, Michitaka Nasu

**Affiliations:** ^1^ Department of Emergency and Critical Care Medicine Urasoe General Hospital Okinawa Japan; ^2^ Department of Thoracic Center Urasoe General Hospital Okinawa Japan

**Keywords:** Aorto‐pleural fistula, bronchial occlusion, massive hemoptysis, Endobronchial Watanabe Spigot

## Abstract

Aorto‐pleural fistula (APF) is a rare, potentially fatal condition that should be immediately treated by an endovascular or surgical approach. In this case, we treated APF using bronchial occlusion with Endobronchial Watanabe Spigots (EWSs) after one‐lung ventilation. Notably, EWS is composed of silicon for endobronchial occlusion under bronchoscopy. An 88‐year‐old man was referred to our hospital for sudden massive hemoptysis. We maintained the airway by emergent intubation into the right main bronchus through guided bronchoscopy. Computed tomography demonstrated an aortic aneurysm at the aortic arch, penetrating the upper lobe of the left lung. On the 18th hospital day, we performed prophylactic endobronchial occlusion with EWS. The patient was extubated shortly thereafter. Endobronchial occlusion with EWS might be effective in patients with APF who exhibit generally poor conditions. Endobronchial occlusion treatment should be performed after controlling massive bleeding by one‐lung ventilation.

## Introduction

Aorto‐pleural fistula (APF) is a rare, critical complication of aortic aneurysm. Notably, it should be managed by an endovascular and/or surgical approach because of the high risk of haemorrhagic shock or massive hemoptysis associated with fatal outcomes.

Endobronchial Watanabe Spigots (EWSs) (Novatech, Grasse, France) are silicone‐made bronchial fillers that were developed in Japan by Watanabe 1. Bronchial occlusion with EWS has been reported as a tool to stop air leakage. The applications of EWS include intractable pneumothorax, pyothorax with bronchial fistula, traumatic hemo‐pneumothorax, and post‐surgical bronchial fistula 1. Recently, bronchial occlusion with EWS has been applied in cases of massive hemoptysis with favourable outcomes 2. Here, we report a case of APF treated by endobronchial occlusion with EWS after emergent unilateral intubation to maintain the airway.

## Case Report

**Figure 1:**
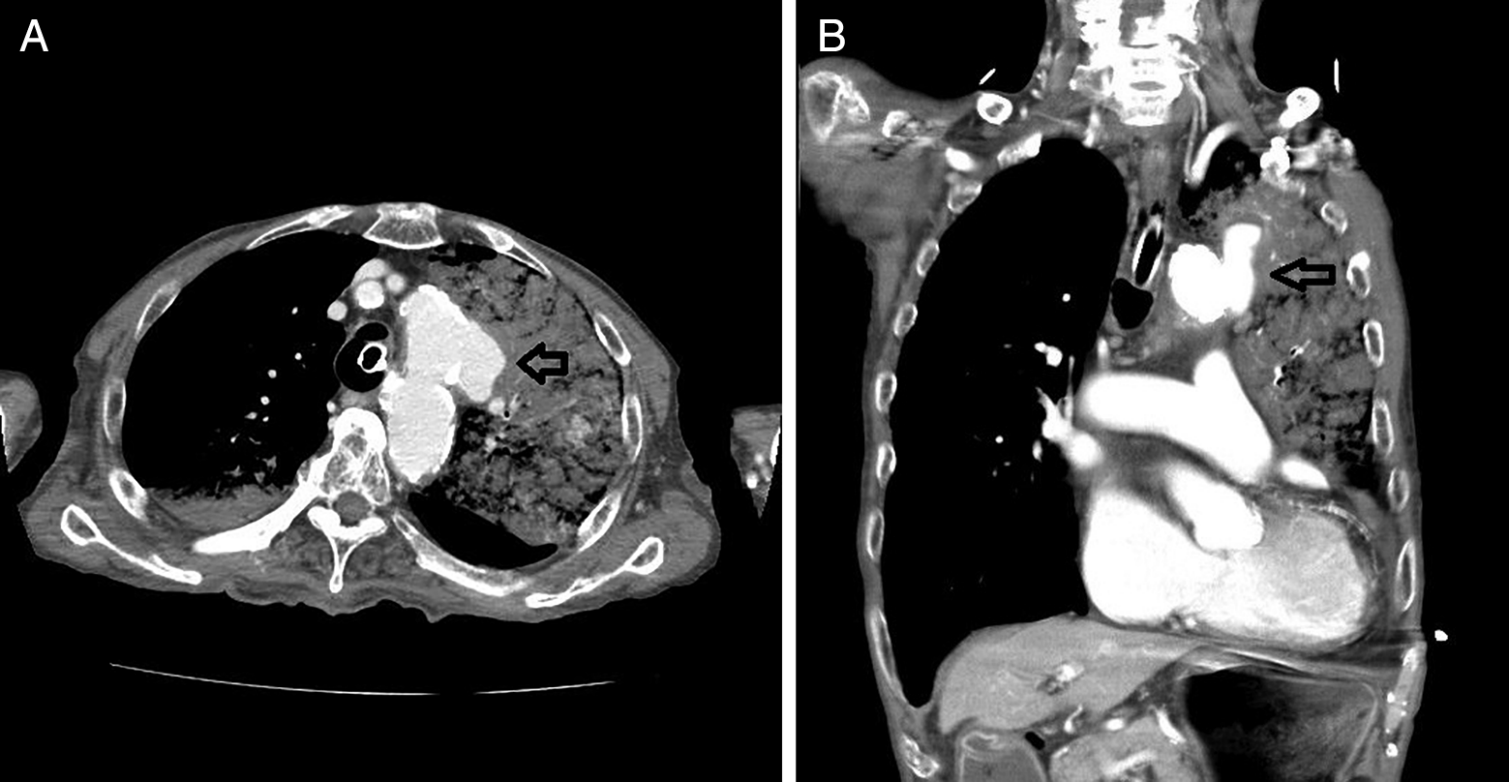
Contrast computed tomography showed an aortic aneurysm at the aortic arch that penetrated the upper lobe of the left lung; blood flowed into the upper lobe of the left lung. (A) Axial view; (B) coronal view.

**Figure 2:**
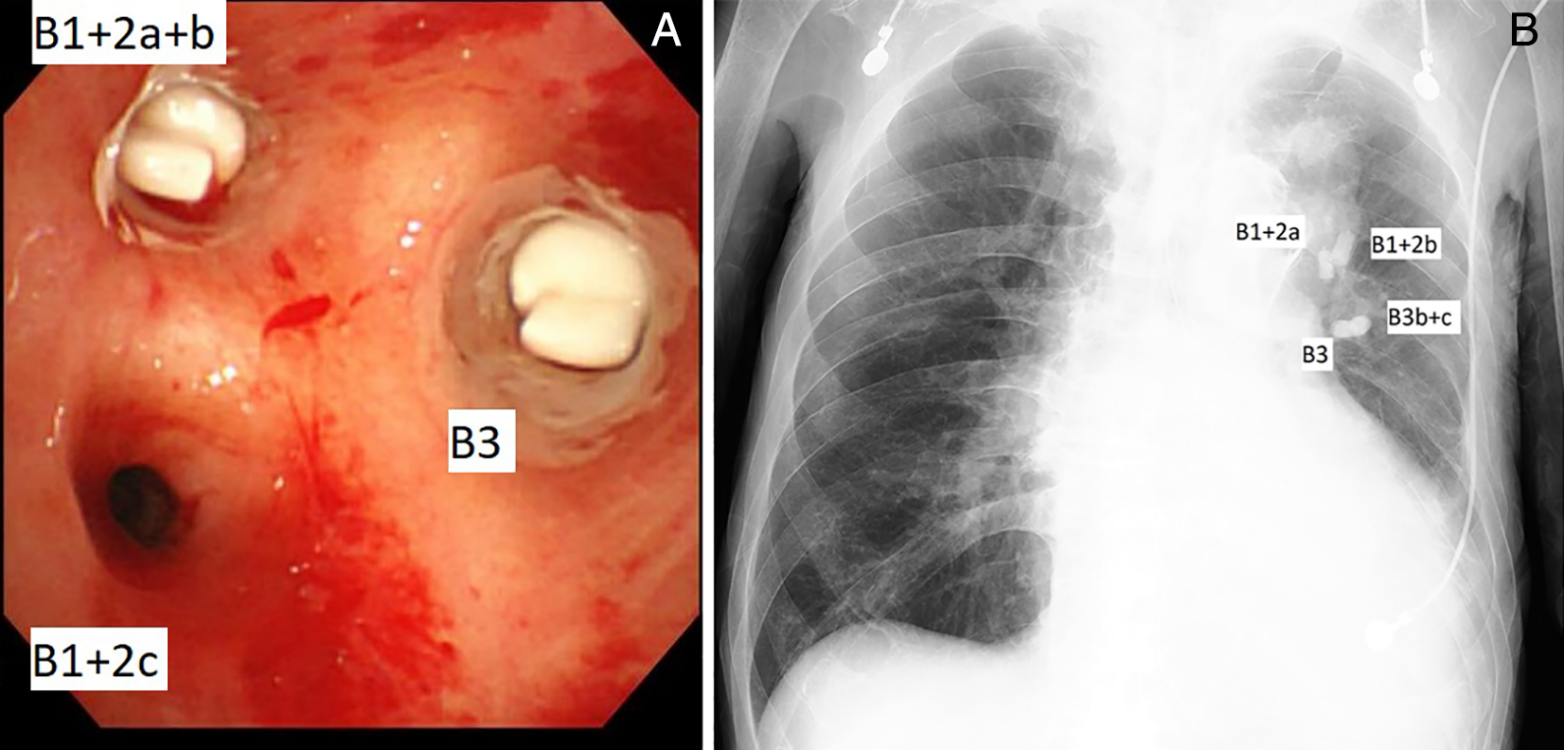
(A) Bronchoscopy showed that Endobronchial Watanabe Spigot (EWS) was inserted into the responsible bronchi in the left lung. In the upper lobe of the left lung, except B1 + 2c, bronchi were filled with EWS. (B) Radiography showed that four EWSs were inserted into each responsible bronchus. Inserted EWSs were all 7 mm in size.

An 88‐year‐old man was referred to our hospital for emergent massive hemoptysis. His medical history was remarkable for chronic heart failure, moderate mitral regurgitation, atrial fibrillation, and chronic kidney disease. He was undergoing treatment with apixaban and pilsicainide for atrial fibrillation. He was severely hypoxic (SpO_2_: 80 under O_2_ 15 L/min by oxygen mask) and hypotensive (systolic blood pressure: 80 mmHg) on admission. In the emergency department, we performed intubation into the right main bronchus through guided bronchoscopy; this was followed immediately by right side one‐lung ventilation as portable chest radiography showed consolidation in the left upper lung. Bronchoscopy showed that the trachea was almost obstructed by haemorrhage and haematoma. He experienced cardiopulmonary arrest immediately after the airway was maintained. However, spontaneous circulation was restored by cardiopulmonary resuscitation. Contrast computed tomography (CT) demonstrated an aortic aneurysm at the aortic arch, which penetrated the upper lobe of the left lung (Fig. 1A,B). We suspected that it would be difficult to perform emergent surgery because of the patient’s poor general condition. Furthermore, we believed that there was no indication for endovascular stenting due to the following reasons: (1) the root of the left brachiocephalic artery was close to the penetrating portion of the aneurysm, at a distance of 12 mm. Thus, there might have been a high risk of obstructing blood flow to the brachiocephalic artery; (2) a risk of aortic injury might have been induced by stenting because the aortic arch was highly calcified. He was admitted to the intensive care unit, and we controlled blood pressure using nicardipine and discontinued anticoagulation therapy and performed platelet and fresh frozen plasma (FFP) transfusion for haemostasis. Bleeding from the APF decreased gradually due to astriction by haematoma. On the 17th hospital day, we performed bronchoscopy for the suction of haematoma, except in the bronchi of the left upper lobe, and adjusted the intubation tube for conversion to bilateral lung ventilation. On the 18th hospital day, we performed endobronchial occlusion with EWS to prevent fatal hemoptysis despite the risk of atelectasis. We inserted the EWS into each target bronchus with haematoma, with consideration of the risk of re‐bleeding due to the removal of the haematoma. EWS sizes were as follows: (B1 + 2a: 7 mm; B1 + 2b: 7 mm; B3b + c: 7 mm; and B3: 7 mm) (Fig. 2A). B1 + 2c did not undergo EWS insertion because this bronchus was not responsible for bleeding on CT findings. There was no massive hemoptysis after bronchial occlusion with the EWS; subsequent mild hemosputum was controlled by a haemostatic drug. The patient was successfully extubated on the 22nd hospital day and was discharged on the 47th hospital day without complications and free from oxygen. Radiography showed that EWS had promptly fixed each bronchus (Fig. 2B).

## Discussion

APF is an abnormal communication between the aorta and lung. It is a very rare and life‐threatening condition, inducing airway obstruction due to massive hemoptysis, haemorrhagic shock, or sepsis. It should be emergently managed by an endovascular and/or surgical approach. It is mainly caused by a thoracic aortic aneurysm with lung infection, pressure necrosis, atherosclerosis, trauma, or previous vascular surgery. In this case, a rapidly progressive thoracic aneurysm might have caused the APF because the patient had no history of traumatic or surgical events, and a CT scan 1 year prior had demonstrated a normal aortic arch.

Patients with APF are often in a critical condition. Clinicians must first protect the airways, preventing asphyxia, and control bleeding by selective one‐sided main bronchial intubation. As definitive interventions, surgical and/or endovascular treatment should be performed immediately. In addition, thick lung wedge resection was reported to be effective 3. This procedure divides the portion of the lung penetrated and the central side of the lung in order to surgically control massive hemoptysis.

In this case, we judged that these surgical interventions were too invasive because of the poor general condition of the patient. We attempted bronchial occlusion with EWS as a less‐invasive approach. EWS was reported as useful and safe for intractable pneumothorax and/or pulmonary fistula, especially for patients with poor general conditions 1. There are few case reports showing that bronchial occlusion with EWS could serve as radical management of massive hemoptysis; notably, there has been no such report for the management of APF 4. Therefore, this is the first report to show that endobronchial occlusion with EWS is effective as a less‐invasive therapy for APF in a patient with poor general condition, although this does not constitute radical therapy. In addition, bronchial occlusion with EWS could not be performed during active massive hemoptysis because a clear field of vision was not accessible under bronchoscopy. Therefore, one‐sided lung ventilation was necessary to reduce endobronchial bleeding by astriction.

## Disclosure Statement

Appropriate written informed consent was obtained for publication of this case report and accompanying images.
